# Phylogenetic and Morphologic Analyses of a Coastal Fish Reveals a Marine Biogeographic Break of Terrestrial Origin in the Southern Caribbean

**DOI:** 10.1371/journal.pone.0011566

**Published:** 2010-07-13

**Authors:** Ricardo Betancur-R, Arturo Acero P., Hermann Duque-Caro, Scott R. Santos

**Affiliations:** 1 Department of Biological Sciences, Auburn University, Auburn, Alabama, United States of America; 2 Universidad Nacional de Colombia sede Caribe, CECIMAR/INVEMAR, Cerro Punta Betín, Santa Marta, Colombia; 3 Duque Caro y Cia. Ltda., Bogotá, Colombia; University of Otago, New Zealand

## Abstract

**Background:**

Marine allopatric speciation involves interplay between intrinsic organismal properties and extrinsic factors. However, the relative contribution of each depends on the taxon under study and its geographic context. Utilizing sea catfishes in the *Cathorops mapale* species group, this study tests the hypothesis that both reproductive strategies conferring limited dispersal opportunities and an apparent geomorphologic barrier in the Southern Caribbean have promoted speciation in this group from a little studied area of the world.

**Methodology/Principal Findings:**

Mitochondrial gene sequences were obtained from representatives of the *Cathorops mapale* species group across its distributional range from Colombia to Venezuela. Morphometric and meristic analyses were also done to assess morphologic variation. Along a ∼2000 km transect, two major lineages, *Cathorops* sp. and *C. mapale*, were identified by levels of genetic differentiation, phylogenetic reconstructions, and morphological analyses. The lineages are separated by ∼150 km at the Santa Marta Massif (SMM) in Colombia. The northward displacement of the SMM into the Caribbean in the early Pleistocene altered the geomorphology of the continental margin, ultimately disrupting the natural habitat of *C. mapale*. The estimated ∼0.86 my divergence of the lineages from a common ancestor coincides with the timing of the SMM displacement at ∼0.78 my.

**Main Conclusions/Significance:**

Results presented here support the hypothesis that organismal properties as well as extrinsic factors lead to diversification of the *Cathorops mapale* group along the northern coast of South America. While a lack of pelagic larval stages and ecological specialization are forces impacting this process, the identification of the SMM as contributing to allopatric speciation in marine organisms adds to the list of recognized barriers in the Caribbean. Comparative examination of additional Southern Caribbean taxa, particularly those with varying life history traits and dispersal capabilities, will determine the extent by which the SMM has influenced marine phylogeography in the region.

## Introduction

Whereas there is tremendous evidence documenting the processes promoting isolation, and ultimately speciation, in terrestrial and freshwater organisms, how such mechanisms operate in marine habitats can be puzzling [1], [2], [3]. Generally, allopatric speciation models are difficult to invoke for marine organisms given the limited opportunities for geographic isolation in a continuous environment and an elevated potential for dispersal due to pelagic broadcast spawning [1], [4]. Furthermore, extrinsic factors such as circulation patterns, temperature regimes, and coastal geomorphology may act as barriers restricting gene flow in marine environments [2], [5], [6]. While such barriers generally provide an avenue for inferring historical vicariant events, only a few regions have been comprehensively examined in this context [7], [8], [9], [10]. On the other hand, intrinsic organismal properties such as limited dispersal abilities also have a strong influence on population structure [11], [12], [13], so that brooders and/or species that undergo direct development are more prone to geographic isolation and genetic segregation than pelagic dispersers [10], [14], [15].

Well studied marine systems with low vagility revealing highly structured populations or deep phylogeographic breaks include the Spiny Damselfish (*Acanthochromis polyacanthus*), the Banggai Cardinalfish (*Pterapogon kauderni*), the Surfperchs (*Embiotica* spp.), the Tidewater Goby (*Eucyclogobius newberryi*), a sea cucumber (*Cucumaria pseudocurata*), the Rock Whelk (*Nucella emarginata*), and the Bamboo Worm (*Clymenella torquata*), among others [12], [16], [17], [18], [19]. However, studying the genetic structure of additional species with limited dispersal abilities can provide further insights into the mechanisms driving marine allopatric speciation as well as the relative contribution of extrinsic and intrinsic factors to this process. Likewise, emphasizing the sampling of understudied areas, in conjunction with phylogenetic and phylogeographic assessments, can help to provide a better understanding of regional patterns in marine biogeography.

The Mapalé Sea Catfish, or *Cathorops mapale* species group, inhabits coastal lagoons and inshore marine waters in the Southern Caribbean [20], [21]. Like other sea catfishes, the *C. mapale* group practices oral incubation and lacks pelagic larval stages. This specialized reproductive mode, coupled with their demersal habits, results in low dispersal capabilities and high rates of species endemism for sea catfishes [22]. Thus, the *C. mapale* group offers an excellent opportunity for identifying potential processes promoting allopatric speciation in the sea. The Mapalé Sea Catfish encompasses two major lineages: *Cathorops mapale sensu stricto*, distributed along the central and southwestern coasts of the Colombian Caribbean, and *Cathorops* sp., occurring from northeastern Colombia through Venezuela [20], [23]. A similar break in faunal composition has been reported for other marine organisms in the region including mollusks and fishes ([24], [25], see Discussion). However, no historical scenarios have been proposed toward explaining this biogeographic pattern.

In light of the above, the present study infers that the limited dispersal opportunities offered by its reproductive strategies, in conjunction with extrinsic factors like geomorphological barriers, have promoted marine allopatric speciation in the *Cathorops mapale* group. To test this, phylogenetic analyses were performed on mitochondrial gene sequences collected along their distributional range. Additionally, divergence times were estimated via molecular clock analyses and morphological variation quantified using morphometric and meristic approaches. Based on these results, we hypothesize that a major barrier on the northern Colombian coast likely promoted allopatric speciation in the *Cathorops mapale* group at the end of the early Pleistocene. In this context, this study provides new insights into the biogeography of the Southern Caribbean, a highly diverse yet understudied area of the world [26].

## Materials and Methods

### Sampling, DNA sequence data, and genetic variation

**Figure 1:**
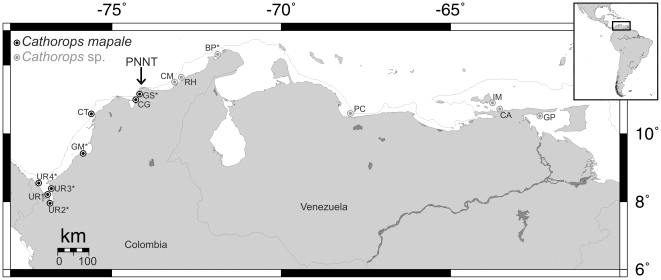
Sampling localities for the *Cathorops mapale* group along the southern Caribbean. Arrow indicates Parque Nacional Natural Tayrona (PNNT), where the continental shelf is narrower (gray line shows 200 m isobath). UR, Urabá; GM*, Golfo de Morrosquillo; CT, Cartagena; CG, Ciénaga Grande de Santa Marta; GS, Golfo de Salamanca; CM, Camarones; RH, Riohacha; BP*, Bahía Portete; PC, Puerto Cabello; IM, Isla Margarita; CA, Carupano; GP, Golfo de Paria (map from www.aquarius.ifm-geomar.de). *Only morphological material examined from these localities.

Taxonomic sampling within the genus *Cathorops* was designed following the phylogenetic hypotheses of Betancur-R. et al. [20] and Betancur-R [27]. In addition to the *C. mapale* group (*C. mapale* and *Cathorops* sp.), the ingroup included the closely related *C. fuerthii* group (*C. fuerthii*, *C.* aff. *fuerthii*, and *C. manglarensis;* from the Eastern Pacific) and *C.* cf. *higuchii* (from Nicaraguan Caribbean). We used *C. spixii*, *C. agassizii*, and *C. hypophthalmus* as outgroups. Sample size within the *C. mapale* group consisted of 17 individuals from each lineage collected at 10 locations along its distributional range, with a focus on neighboring localities from either side of the Parque Nacional Natural Tayrona (PNNT) in Santa Marta, Colombia (Fig. 1; Table S1), which represents the distributional breakpoint between the lineages (see below). This sample size represents individuals collected during multiple field trips to Venezuela and Colombia from 2003 to 2008 by RBR and AAP. Institutional abbreviations are as listed at ASIH website (2010) http://www.asih.org/codons.pdf, with the addition of stri-x: tissue collection, Smithsonian Tropical Research Institute. SL is standard length. Two letter country codes follow ISO-3166.

Targeted mitochondrial regions included partial *cytochrome b* (*cyt b*) and the complete *ATP synthase* subunits 8 and 6 (*ATPase 8/6*) protein-coding genes. Nucleic acid extractions, PCR conditions, utilized primers, and sequence alignment procedures are as described in Betancur-R. et al. [23]. The software DnaSP v. 5 [28] was used to estimate haplotype diversity as well as levels of sequence polymorphism. Corrected genetic distances were calculated in PAUP* v.4.0b10 [29].

### Phylogenetic reconstructions

Phylogenetic reconstructions were performed under maximum likelihood (ML), Bayesian inference (BI), and maximum parsimony (MP) criteria. For ML and BI, the number of model parameters was estimated using the Akaike information criterion (AIC) in ModelTest v. 3.7 [30]. The ML analyses were performed in Garli v. 0.96 [31] with ten runs from random-starting seeds to ensure convergence of likelihood scores. Model parameters were estimated simultaneously (i.e., unfixed) and remaining settings left at default values. The ML nodal support was assessed using the fast bootstrapping algorithm via automatic estimation of runs in RAxML [32] as implemented in the CIPRES portal v.1.15 (2010) http://www.phylo.org/.

The BI analyses were performed in MrBayes v.3.1.2 [33] via Markov chain Monte Carlo (MCMC) iterations. The MCMC searches were conducted in triplicate using four chains. Each search was run for 4.0×10^6^ generations, with tree sampling every 100 generations. Ten percent of the initial trees sampled in each MCMC run were discarded as burn-in. To confirm that post-burn-in trees represent the actual MCMC posterior distribution, marginal parameters (i.e., the MrBayes log file) were analyzed using the Effective Sample Size (ESS) statistic in the program Tracer [34]. ESS values greater than 200 were obtained for all parameters, suggesting that the MCMC searches were run for a sufficient duration to accurately represent the posterior distribution [34]. The post-burn-in samples of the three independent runs were combined in order to estimate marginal probabilities of summary parameters, consensus phylograms, and posterior probabilities of nodes. The MP reconstructions were conducted in PAUP* via heuristic searches with random addition of sequences (10000 replicates) and the tree-bisection-reconnection algorithm.

### Divergence time estimations

**Figure 2:**
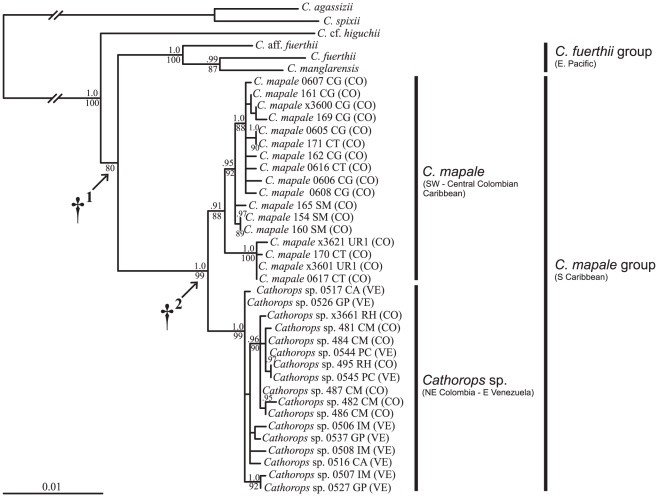
Phylogenetic hypothesis for the *Cathorops mapale* group and related species inferred from mitochondrial sequences (∼2 kbp). Phylogram shown was estimated from ML analyses (lnL -4686.70); well-supported clades are congruent with MP and BI topologies (outgroup *Cathorops hypophthalmus* not shown). Numbers below and above nodes represent RAxML bootstrap values (300 replicates via automatic estimation of runs) and Bayesian posterior probabilities, respectively (well-supported clades only). †^1^ Molecular clock calibration point: Pliocene rising of Panama isthmus. †^2^ Molecular clock estimation point for testing the hypothetical vicariant event separating *Cathorops* sp. and *C. mapale*: northward displacement of Santa Marta Massif and disruption of continental shelf (end of early Pleistocene). Locality abbreviations follow Fig. 1 and Table S1 (ISO-3166 country codes given in parenthesis).

Molecular clock analyses were performed to infer the divergence time for the *Cathorops* sp./*C. mapale* stem node (Fig. 2: †^2^). Two different methods were conducted to assess rate heterogeneity among sequences: relative rate tests (RRT) based on likelihood, as implemented in the software r8s v.1.71 [35], [36], and likelihood ratio tests (LRT) as implemented in PAUP*. Both tests failed to reject the null hypothesis of clock-like behavior (see Results); thus, divergence times were estimated under the assumption of a molecular clock via the likelihood-based Langley-Fitch (LF) method in r8s [36]. For clock calibration, the final rise of the Panama isthmus (3.1–2.8 mya [37]) was utilized as the hypothetical vicariant event leading to the divergence of the *Cathorops mapale* (Southern Caribbean) and *C. fuerthii* (Eastern Pacific; node †^1^, Fig. 2) groups from a common ancestor. The mitochondrial distances calculated from protein-coding sequences between the two groups (2.2–2.8% [20]) are similar to those reported for other transisthmian fish pairs assumed to have diverged during the final rise of the isthmus [7], [38]. Both maximum and minimum age constraints (3.1 and 2.8 my, respectively) were applied to node †^1^ (see also [27]).

### Morphometric and meristic analyses

Morphological variation within the *Cathorops mapale* group was quantified using morphometric and meristic analyses. Measurements were taken with either a ruler and recorded to the nearest millimeter (mm) or with dial callipers and recorded to the nearest 0.1 mm. Thirty five measurements representing truss homologous points were made on 18 individuals of *Cathorops mapale* and 20 individuals of *Cathorops* sp. (Text S1). Twenty-eight measurements were recorded as specified in Betancur-R. (2007); the remaining seven follows Marceniuk (2007) (Text S2). Principal component (PC) analyses were conducted to estimate size-free shape variation by reducing the dimensionality of the dataset while retaining as much variation as possible (Jolliffe, 2002). The PC analyses were performed on a covariate matrix of log-transformed measurements in the software JMP (SAS Institute). Univariate analyses were conducted to find potential morphometric differences between the two lineages by plotting two measurements with opposite polarity (as identified by PC analyses).

Counts for meristic analyses were obtained from fins (pectoral-fin and anal-fin rays) and gill arches (gill rakers on first and second arches) on 18 individuals of *Cathorops mapale* and 24 individuals of *Cathorops* sp. All counts included rudimentary elements and the best meristic discriminators were arranged into a frequency table.

### Ethics statement

All animals were handled in strict accordance with good animal practice as defined by the relevant local animal welfare bodies; the institutions involved approved animal work.

## Results

### Sequence analyses and genetic variation

The mitochondrial protein-coding gene sequences utilized here are available from GenBank under the accession numbers listed in Table S1. The final alignment included 1937 bp, with 1095 bp coming from the partial *cyt b* and 842 bp for the complete *ATPase 8/6* (see also [23], [27]). In the concatenated alignment, 31 positions had missing data due to ambiguity in the chromatogram reads; these were excluded from the genetic differentiation analyses. As previously suggested [20], measures of genetic differentiation, phylogenetic reconstructions, and morphological analyses (but see below) support two major lineages within the *Cathorops mapale* group: *C. mapale*, encompassing individuals from Urabá (southwestern Colombian Caribbean) through Santa Marta (central Colombian Caribbean; localities UR, GM, CT, CG, GS; see details and abbreviations in Fig. 1) along a ∼450 km of coastline. On the other hand, individuals from an ∼1400 km of coastline from Riohacha (northern Colombian Caribbean) through Golfo de Paria (eastern Venezuelan; localities CM, RH, BP, PC, IM, CA, and GP; Fig. 1) belong to *Cathorops* sp. The two lineages are separated by at least ∼150 km, with the PNNT situated along this particular stretch of coastline (Fig. 1).

Measures of sequence variation within each lineage and overall are summarized in Table 1. Polymorphisms between sequences were confined to point mutations, with an absence of nucleotide insertions or deletions. Notably, no haplotypes were shared between *Cathorops mapale* and *Cathorops* sp. or between geographic locations separated by the PNNT (see above). The two lineages were also distinguishable by eight fixed substitutions, three in *cyt b* and five in *ATPase 8/6*. Haplotype diversity was high, with similar values obtained from *C. mapale* (0.978) and *Cathorops* sp. (0.985). *Cathorops mapale* possessed higher values for polymorphic sites (PS = 31) and parsimony-informative sites (PIS = 15) than *Cathorops* sp. (PS = 23; PIS = 10). Corrected genetic distances (based on a GTR+I+Γ model, see below) among lineages of the *C. mapale* group were on average higher (0.71–1.23%) than within groups (0–0.82% for *C. mapale*, 0–0.53% for *Cathorops* sp.).

**Table 1:** Summary of sequence variation statistics and genetic distances for the *Cathorops mapale* group.

Source	HD	PS	PIS	GD (GTR+I+Γ)
*Cathorops mapale*	0.978	31	15	0–0.0082
*Cathorops* sp.	0.985	23	10	0–0.0053
Overall (*C. mapale* group)	0.991	60	33	0.0071–0.0123

HD, haplotype diversity.

PS, polymorphic sites.

PIS, parsimony-informative sites.

GD, genetic distances.

### Phylogenetic analyses and divergence time estimations

All phylogenetic reconstructions were performed on the concatenated dataset containing 1937 bp (Fig. 2). Both ML and BI analyses were conducted under a GTR+I+Γ model as selected by the AIC and a single partition. The ML (optimal tree score =  lnL −4686.70), BI (mean posterior probability score =  lnL −4978.81), and MP (24 optimal trees of 378 steps) reconstructions resulted in highly congruent topologies. Although a few poorly supported nodes within *Cathorops* sp. or *C. mapale* were in disagreement among the different reconstruction methods, all species-level clades were identical and well supported. As suggested by previous studies [20], [23], [27], the *C. fuerthii* group from the Eastern Pacific was recovered as the sister clade of the *C. mapale* group and *Cathorops sp.* and *C. mapale* were reciprocally monophyletic in all analyses (Fig. 2).

The RRT performed with different nesting hierarchies on the three clades failed to reject the null hypothesis of clock-like behavior (χ^2^ = 0.08–0.49; d.f. = 1; *p* = 0.48–0.77). Similarly, the LRT suggested no significant rate heterogeneity when comparing the likelihood scores of clock-enforced and non-enforced optimizations on a neighbor-joining tree calculated with the model parameters obtained from ModelTest (outgroup *Cathorops hypophthalmus* excluded from the analyses; χ^2^ = 33.3; d.f. = 38; *p* = 0.68). The LF method estimated that the split between *Cathorops* sp. and *C. mapale* occurred 0.89 my ago, with a substitution rate of 0.56%/my/lineage.

### Morphometric and meristic analyses

**Figure 3:**
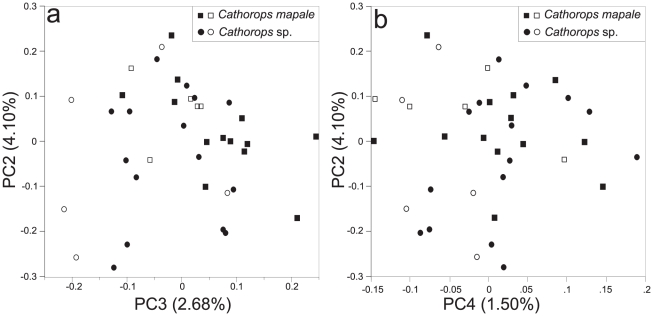
Principal component analysis of 35 morphometric variables from the *Cathorops mapale* group. Scatterplots of (A) PC2 vs. PC3 and (B) PC2 vs. PC4 (percent of variation for each PC given in parenthesis). Opened and filled symbols represent males and females, respectively.

**Figure 4:**
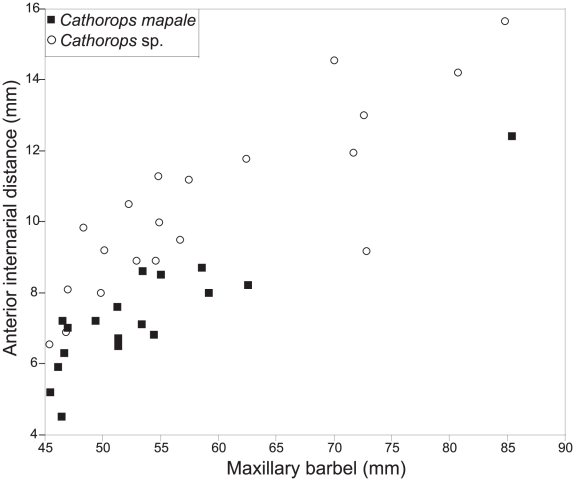
Best morphometric discriminator for the *Cathorops mapale* group. Plot of maxillary barbel vs. posterior internarial distance.

In the PC analysis, PC1, PC2, PC3, and PC4 explained 87.59%, 4.10%, 2.68%, and 1.50% of the variation, respectively. While PC1 is the size factor, the remaining components represent size-free shape variation [39]. Scatterplots of PC2 vs. PC3 and PC2 vs. PC4 revealed morphometric overlap for *Cathorops* sp. and *C. mapale* (Fig. 3). Similar results were obtained after removing 18 morphometric variables (see Text S2) potentially associated with sexual dimorphism in *Cathorops* (results not shown, [21], [40]). Furthermore, males and females overlapped in all analyses, suggesting morphometric variation is not mainly driven by sexual differentiation. Despite the observed overlap in the multivariate analyses, *Cathorops* sp. and *C. mapale* were separated by the averages of a morphometric ratio and the modes of two meristic variables (although some overlap occurs). The bivariate plot of maxillary barbel vs. posterior internarial distance was the best morphometric discriminator (Fig. 4; maxillary barbel/posterior internarial distance: 4.8–7.9, mean 6.1± SD 0.8 in *C. mapale*; 3.6–6.0, mean 4.4± SD 0.8 in *Cathorops* sp.). For the meristic analyses, anterior rakers on first (20–24, mode 23, in *C. mapale*; 16–21, mode 18, in *Cathorops* sp.) and second (20–24, mode 23, in *C. mapale*; 16–21, mode 18, in *Cathorops* sp.) gill arches were the best variables differentiating the two lineages (see details in Table 2).

**Table 2:** Frequency table summarizing best meristic variables differentiating lineages within the *Cathorops mapale* group.

**1st arch upper limb**	**4**	**5**	**6**	**7**	**8**					**n**
*Cathorops* sp.	1	9	14							24
*C. mapale*			5	10	3					18
**1st arch lower limb**	**12**	**13**	**14**	**15**	**16**					**n**
*Cathorops* sp.	6	13	4	1						24
*C. mapale*			1	5	12					18
**1st arch total**	**16**	**17**	**18**	**19**	**20**	**21**	**22**	**23**	**24**	**n**
*Cathorops* sp.	1	3	8	7	4	1				24
*C. mapale*					1	3	3	8	3	18
**2nd arch lower limb**	**11**	**12**	**13**	**14**	**15**					**n**
*Cathorops* sp.	2	5	9	5						21
*C. mapale*			1	9	7					17
**2nd arch total**	**16**	**17**	**18**	**19**	**20**	**21**				**n**
*Cathorops* sp.	2	5	7	6	1					21
*C. mapale*			1	4	11	1				17

## Discussion

Compared to the terrestrial environment, a relative small number of barriers that may promote allopatric speciation in marine organisms have been documented [1]. Furthermore, defining biogeographic breaks at regional scales may be complex as a result of the varying dispersal capabilities among marine organisms [9], [10]. This study provides evidence for a biogeographic break apparently responsible for allopatric speciation in a coastal Neotropical fish lacking pelagic larval stages. Phylogenetic hypotheses derived from mitochondrial gene sequences revealed a deep break for the *Cathorops mapale* group around the PNNT in northern Colombia (Fig. 1). While multivariate and frequency-data analyses reveal either full or partial overlapping between *C. mapale* and *Cathorops* sp. at the morphological level (Figs. 3, 4; Table 2), the two lineages show complete segregation at the mitochondrial level (Fig. 2).

The reciprocal monophyly and eight fixed substitutions observed between *Cathorops* sp. and *C. mapale* suggest that, once established (ca. 0.8 mya, see below), the isolating barrier was maintained and effectively restricted gene flow across the PNNT from that point through to the present time. Although some mixing between the lineages near the barrier's boundary cannot be completely ruled out, our sampling of individuals from locations adjacent to the boundary does not provide support for this scenario. Further sampling around the boundary region, as well as comparative phylogeographic studies utilizing other marine organisms with low dispersal capabilities (e.g., other sea catfishes, toadfishes, gobies, gastropods), are crucial toward describing the extent to which the suggested biogeographic break may be restricting gene flow and promoting allopatric speciation in the marine environment (see below).

On the other hand, male-mediated gene flow might provide an alternative interpretation to allopatric speciation given the matrilineal segregation and partial morphological overlap reported here. In this scenario, although females with restricted migration would promote divergence of the mitochondrial genome, male-biased migration could facilitate the transport of nuclear genes across the barrier while simultaneously impeding phenotypic differentiation. Although this study examined no nuclear markers to test this competing hypothesis, we feel this is an unlikely situation for multiple reasons. First, compelling evidence suggests niche conservatism, and corresponding morphological stationarity, is a common result of allopatric speciation due to a lack of differential selective regimes that might drive morphological divergence (i.e., both *Cathorops* sp. and *C. mapale* retain the ancestral morphological traits that facilitate utilization of a specific niche) [41], [42], [43]. Second, prominent examples of male-mediated gene flow typically involve taxa with long-distance migration and nesting-site fidelity, such as the green sea turtle (*Chelonia mydas*) that migrates between foraging and nesting locations separated by hundreds to thousands of kilometers [44], [45]. While studies on sea catfish biology have documented subtle seasonal migration between adjacent habitats (e.g., [46], [47]), to our knowledge, no evidence of extensive migratory behaviors have been reported. Moreover, given that male sea catfishes (including the *Cathorops mapale* group) practice oral incubation, suggesting both sexes invest high energetic resources into reproduction (e.g., [48]), male-biased migratory behavior (i.e., vagile males and sedentary females) seems implausible. Lastly, coincidental patterns of similar distributions and regional endemism documented for many other fish as well as invertebrate species in the area reinforce the allopatric hypothesis we propose for the *Cathorops mapale* group (see below).

### Caribbean biogeography and the Santa Marta Massif

Given the apparent absence of barriers to gene flow, marine biogeographers have long questioned whether Caribbean populations are genetically homogeneous or geographically segregated. Although many species are widely distributed in the Greater Caribbean, regional endemism has traditionally suggested the presence of biogeographic breaks, such as in the Florida peninsula [49], the West Indies [50], the Bahamas [51], and the Southern Caribbean [25], [52], [53]. In the 90's, Shulman and Bermingham [5] examined mitochondrial restriction fragment length polymorphisms (RFLPs) of fish species with varying dispersal mechanisms, concluding that Caribbean populations were widely interconnected. However, within the past decade, studies using biophysical models and more sensitive molecular techniques on broader taxonomic arrays have revealed regional subdivision within the Caribbean and phylogeographic breaks in several species, including gobies, serranids, damselfishes, and acroporid corals [54], [55], [56], [57]. Generalized trends from these studies imply isolation of the Florida peninsula from the rest of the Greater Caribbean and biogeographic breaks around the central Bahamas and the Mona Passage between Hispaniola and Puerto Rico. Likewise, the Amazon barrier in northeastern South America, formed by the outflow of the Amazon and Orinoco Rivers, has been shown to play an important role in the formation of numerous geminate pairs between Brazilian and Caribbean shallow reef faunas (e.g., [58], [59]).

Although much attention has been paid toward documenting biogeographic trends in the Western Atlantic, patterns and processes shaping the distribution of marine organisms in the Southern Caribbean, where the *Cathorops mapale* group occurs, remain poorly understood. Notably, the continental shelf of the Southern Caribbean, roughly extending from Costa Rica to the Orinoco delta [25], is considered one of two hotspots (along with the East Indies triangle) for marine biodiversity in the world [26]. Based on mollusk faunal composition and endemism, the Southern Caribbean has been long identified as being isolated from the rest of the Caribbean and otherwise more allied with the Eastern Pacific (e.g., [25], [53], [60]). Although the delimitation of biogeographic units in the Southern Caribbean has been debated, most studies recognize a Colombian–Venezuelan–Trinidad (CVT) subprovince, extending from around Santa Marta northeastwards to Trinidad. Some species, such as the gastropods *Voluta musica* and *Phyllonotus margaritensis* [24], [25], and the fishes *Paralabrax dewegeri*, *Citharichthys minutus*, *C. valdezi*, as well as *Cathorops* sp. (A. Acero P., unpubl. data; [61]), are chiefly confined to the CVT subprovince. For many other species whose range partially overlaps the CVT (e.g., *Colomesus psittacus*, *Paralabrax dewegeri*, and *Genyatremus luteus*), their western distributional limit coincides with the Santa Marta-La Guajira boundary, around the PNNT. This abrupt break in faunal composition has been attributed to the combined effects of the narrow coastal shelf and cold up-welling waters influencing the region [24], [25]. While these previous accounts place this regional endemism in a descriptive framework, neither historical scenarios or supporting genetic data explaining such biogeographic patterns and breaks of the Southern Caribbean have to date been reported.

**Figure 5:**
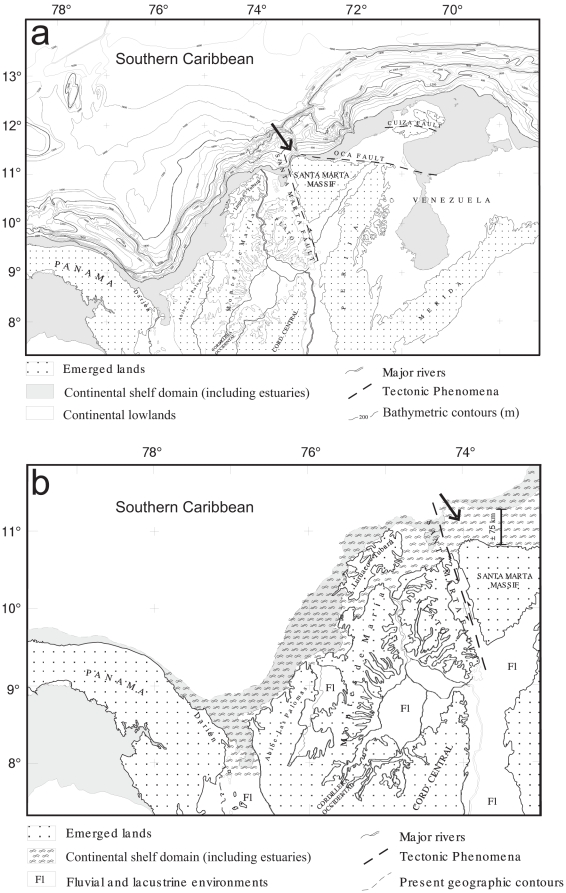
Geographic configuration of Colombia and bathymetric profile of the Southern Caribbean. (A) Present configuration (modified from [72]). (B) Early Pleistocene paleogeographic reconstruction (after [65]). Arrows indicate the narrow vs. wide continental shelf adjacent to the Santa Marta Massif in present vs. early Pleistocene times, respectively.

Based on the data presented here, this study hypothesizes that the basal genetic break in the *Cathorops mapale* group in northern Colombia around the PNNT resulted (in part, see below) from the geological progression of the Santa Marta Massif (SMM;  =  Sierra Nevada de Santa Marta). The SMM is a prominent triangular geomorphic feature of 5800 m elevation facing the Caribbean (Fig. 5). Although there has been much debate regarding its origin and isolated position in northwestern South America, two major transcurrent faults bound the SMM and appear actively associated with its tectonic emplacement history. These are the Oca fault, in the north, and the Santa Marta fault along the northwest (Fig. 5a, [62], [63], [64], [65]). Campbell [62] proposed a 110 km displacement of the SMM to the north to reach its present position during post Miocene times [66]. Duque-Caro [65] estimated an age comprising the Pliocene-Pleistocene boundary, during the time of Andean Orogeny and transcurrent faulting phenomena, such as that of the Santa Marta Fault (see also [67]). Stratigraphic, biostratigraphic and chronostratigraphic reassessments of historic and recent data from the areas surrounding the SMM (H. Duque-Caro and G. Guzmán-Ospitia, in prep.) indicate that the most recent activity of the Santa Marta Fault, which emplaced the SMM to its present position, was in the order of 75 km (Fig. 5b) and took place by the end of early Pleistocene epoch (ca. 0.78 mya). In total, the displacement of the SMM altered the geomorphology in the continental margin of the Colombian Caribbean by disrupting the shelf connection between both the western and eastern sides of the massif, effectively making the shelf narrower and shallower around the PNNT (Fig. 1; Fig. 5a).

Given the above, the major changes in the geomorphological configuration of continental margin along the Colombian Caribbean fragmented the natural soft bottom habitat of *Cathorops* and allowed the formation of coral reef assemblages in the PNNT, leading to the apparent emergence of a barrier to gene flow for the genus. Alternatively, but not mutually excusive, the continental slope might have been exposed during low sea level episodes (as a result of the narrow shelf), causing local extirpation of *Cathorops*. A similar scenario has been suggested to explain the absence of several marine fishes around the ‘hump’ of Brazil [58].

Further support for our hypothesis comes from divergence times inferred via molecular clock analyses. Specifically, the estimate of 0.86 my for the split of *Cathorops* sp. and *C. mapale* closely match those predicted by the most recent geological evidence for the progression of the SMM (end of the early Pleistocene time ∼0.78 my). The application of comparative phylogeographic approaches involving multiple taxa will help to determine the extent to which the SMM-PNNT barrier has shaped coastal biogeography in the Southern Caribbean.

Alternatively, oceanographic scenarios could also explain the observed phylogenetic break in *Cathorops.* For instance, detailed biophysical models by Cowen et al. [56] suggest a discontinuity around La Guajira, a region influenced by strong seasonal upwelling and offshore currents [68]. From their population genetic analyses of the coral *Acropora palmata*, Baums et al. [54] documented a break somewhere between Panama and Venezuela (samples of Colombian *A. palmata* were not included in their analysis), which is reportedly the result of habitat disruptions segregating coral-reef-dwelling and upwelling-tolerant species. Additional investigations of coral populations suggest the freshwater runoff of the Magdalena River (located ∼50 km southwest of PNNT) can sporadically influence marine waters around Santa Marta, leading to the generation of local phylogeographic breaks (J.A. Sanchez pers. comm.). While these oceanographic factors may impact population structure in reef species, we believe that these are unlikely scenarios for interrupting gene flow among continental estuarine taxa with low vagility inhabiting shallow muddy bottoms. Notably, *Cathorops mapale sensu stricto* occurs at either side of the Magdalena delta (genetic samples collected eastwards and westwards from the river were analyzed here), indicating that the river per se is not generating this major biogeographic break. Furthermore, recent examination of marine phylogeographic breaks based on quantitative approaches have revealed that historical processes (e.g., the geological progression of the SMM) are typically responsible for shaping the distribution among poor dispersers (e.g., the *C. mapale* group) whereas contemporary oceanography (e.g., upwelling) is more of a determinant factor for structuring phylogeography in planktonic dispersers [10]. Future work on species with antagonistic life histories (i.e., short vs. long distance dispersal) and habitat preferences (i.e., soft vs. reef bottoms) in the Southern Caribbean will provide a framework to test these predictions.

### Taxonomic implications and conservation aspects

Some evident questions emerge from our study of the *Cathorops mapale* group at the mitochondrial and morphological levels. For example, should *Cathorops* sp. and *C. mapale* be recognized as separate species? Likewise, should the *Cathorops mapale* group comprise a single species with broader circumscription? Meristic and morphopometric analyses all reveal partial overlap between the two lineages. Also, while mitochondrial distances range from 1.5%–2.8% among sister-species pairs in the genus *Cathorops* (as corrected by the Kimura-2-parameter model; see [20]), divergence between *C. mapale* and *Cathorops* is only 0.7–1.2%. Furthermore, divergence time estimates for the split of the two lineages (∼0.9 my) are slightly lower than generally inferred times for allopatric speciation in fishes (2.3–1.0 my, [69]). Considering the incomplete morphological differentiation as well as the comparatively low mitochondrial distances and recent divergence times, we conclude that *C. mapale* and *Cathorops* sp. represent a case of incipient speciation. Nevertheless, in a taxonomic framework, it is appropriate to recognize the specific status of these lineages. This is particularly relevant considering that Mapalé Sea Catfish play an important role in artisanal fisheries for coastal populations along Colombia and Venezuela. Giving that overfishing at localities such as Ciénaga Grande de Santa Marta has lead to a progressive reduction in reported catch size below that of the minimum maturation size in recent years [70], [71], the fishery may require conservation and management in the immediate future.

## Supporting Information

Table S1 Molecular material examined and GenBank accession numbers for the *Cathorops mapale* group. Locality codes follow Fig. 1; two letter country codes follow ISO-3166. See Betancur-R. (2009) for details on outgroup (non-*Cathorops mapale* group) material.
(0.02 MB XLS)

Text S1 Morphological material examined for the *Cathorops mapale* group. Locality abbreviations given in parentheses follow Fig. 1; two letter country codes follow ISO-3166.
(0.03 MB DOC)

Text S2 Morphometric measurements utilized for multivariate analyses of the *Cathorops mapale* group. Asterisk (*) indicate variables potentially associated with sexual dimorphism (Acero P. et al., 2005; Marceniuk & Betancur-R., 2008).
(0.02 MB DOC)
