# Supplementary material for: Phylogenetic and Morphologic Analyses of a Coastal Fish Reveals a Marine Biogeographic Break of Terrestrial Origin in the Southern Caribbean
Source: PLoS One. 2010 Jul 13;5(7):e11566. doi: 10.1371/journal.pone.0011566 (PMC2903491; doi:10.1371/journal.pone.0011566)
Supplement: Text S1 — Morphological material examined for the Cathorops mapale group. Locality abbreviations given in parentheses follow Fig. 1; two letter country codes follow ISO-3166. (0.03 MB DOC) [file pone.0011566.s002.doc]

**Text S1.** Morphological material examined for the *Cathorops mapale* group. Locality abbreviations given in parentheses follow Fig. 1; two letter country codes follow ISO-3166*.*

*Cathorops mapale.* INVEMAR-PEC 5348 (1) (stri-x3601), 118 mm SL, ICN-MHN 8247 (1), 143 mm SL, Mouth of Río Atrato (El Roto), Golfo de Urabá (UR1), Antioquia, CO; INVEMAR-PEC 5732 (1), 157 mm SL, Golfo de Urabá (UR2), Antioquia, CO; INVEMAR-PEC 5731 (2), 139–172 mm SL, Golfo de Urabá (UR3), Antioquia, CO; INVEMAR-PEC 5501 (2), 151–152 mm SL, Golfo de Urabá (UR4), Antioquia, CO; INVEMAR-PEC 3654 (1), 180 mm SL, Ciénaga de Soledad, Golfo de Morrosquillo (GM), CO (9°20’ N, 75°52’ W); INVEMAR-PEC 5197 (1), 156 mm SL, mouth of Río Tinajones, Golfo de Morrosquillo (GM), CO (9°26’ N, 75°57’ W); ICN-MHN 8246 (2), 165–179 mm SL, mouth of Río Sinú, Golfo de Morrosquillo (GM), CO (9°26’ N, 75°55’ W); INVEMAR-PEC 5333 (1, holotype) (stri-x3600), 183.5 mm SL, ICN-MHN 8244 (1), 166 mm SL, Ciénaga Grande de Santa Marta (CG), CO (10°59’ N, 74°17’ W); INVEMAR-PEC 277 (1) 256 mm SL, Tasajera, Grande de Santa Marta (CG), CO (11°0’ N, 74°20’ W); INVEMAR-PEC 1584 (1), 166 mm SL, Costa Verde, near Ciénaga Grande de Santa Marta (CG), CO (11°02’ N, 74°15’ W); ICN-MHN 8245 (2), 164–196 mm SL, INVEMAR-PEC 5730 (1), 157 mm SL, Golfo de Salamanca (GS), CO (11°06’ N, 74°18’19’ W). See details in (Betancur-R. & Acero, 2005).

*Cathorops* sp. INVEMAR-PEC 5734 (1) (stri-x3661; tissue tag 494), 251 mm SL, NE Riohacha (RH), CO (11°35’ N, 72°53’ W); INVEMAR-PEC 5735 (3) (tissue tags 481–482), 218–247 mm SL, Camarones (CM), CO (11°26’ N, 73°05’ W); INVEMAR-PEC 1448 (3), 94–105 mm SL, Bahía Portete (BP), CO; AUM 42848 (2) (tissue tags VEN05044-VEN05045), 218–239 mm SL, Puerto Cabello (PC), VE; AUM 42758 (4) (tissue tags VEN05006-VEN05008), 171–204 mm SL, Isla Margarita (IM), VE; AUM 42846 (4) (tissue tags VEN05016-VEN05017), 144–205 mm SL, Carupano (CA), VE; AUM 42760 (2) (tissue tag VEN05037), 159–265 mm SL, Irapa, Golfo de Paria (GP), VE; AUM 44227 (5) (tissue tags VEN05026–VEN05027), 225–314 mm SL, Güiria, Golfo de Paria (GP), VE.
