# Supplementary material for: Phylogenetic and Morphologic Analyses of a Coastal Fish Reveals a Marine Biogeographic Break of Terrestrial Origin in the Southern Caribbean
Source: PLoS One. 2010 Jul 13;5(7):e11566. doi: 10.1371/journal.pone.0011566 (PMC2903491; doi:10.1371/journal.pone.0011566)
Supplement: Text S2 — Morphometric measurements utilized for multivariate analyses of the Cathorops mapale group. Asterisk (*) indicate variables potentially associated with sexual dimorphism (Acero P. et al., 2005; Marceniuk & Betancur-R., 2008). (0.02 MB DOC) [file pone.0011566.s003.doc]

**Text S2.** Morphometric measurements utilized for multivariate analyses of the *Cathorops mapale* group. Asterisk (*) indicate variables potentially associated with sexual dimorphism (Acero P. *et al.*, 2005; Marceniuk & Betancur-R., 2008).

Following Betancur-R. (2007: fig. 1, table 1): standard length, body width, head length*, head width*, head depth*, snout length*, mouth width*, upper lip width, maxillary barbel, mandibulary barbel, mental barbel, anterior internarial distance*, posterior internarial distance*, interorbital distance*, eye diameter, postorbital length*, predorsal-fin length*, dorsal-fin base, distance between dorsal and adipose fins, preadipose-fin length*, adipose-fin base, prepectoral-fin length*, pectoral-fin base, prepelvic-fin length*, preanal-fin length*, anal-fin base, anal-fin height, and caudal peduncle depth (total: 28 measurements).

Following Marceniuk (2007: fig. 3, table 1): distance from posterior narine to the eye*, skull width between lateral ethmoids*, skull width between frontals*, posterior width of supraoccipital process, length of supraoccipital process, width of nuchal plate, length of nuchal plate (total: seven measurements).
